# Restoring Behavior via Inverse Neurocontroller in a Lesioned Cortical Spiking Model Driving a Virtual Arm

**DOI:** 10.3389/fnins.2016.00028

**Published:** 2016-02-09

**Authors:** Salvador Dura-Bernal, Kan Li, Samuel A. Neymotin, Joseph T. Francis, Jose C. Principe, William W. Lytton

**Affiliations:** ^1^Department of Physiology and Pharmacology, State University of New York Downstate Medical CenterBrooklyn, NY, USA; ^2^Department of Electrical and Computer Engineering, University of FloridaGainesville, FL, USA; ^3^BME Cullen College of Engineering, University of HoustonHouston, TX, USA; ^4^Department of Neurology, State University of New York Downstate Medical CenterBrooklyn, NY, USA; ^5^Department of Neurology, Kings County Hospital CenterBrooklyn, NY, USA

**Keywords:** neurostimulation, spiking network model, biomimetic, kernel adaptive filtering, inverse model, musculoskeletal arm, virtual arm, neuroprosthetics

## Abstract

Neural stimulation can be used as a tool to elicit natural sensations or behaviors by modulating neural activity. This can be potentially used to mitigate the damage of brain lesions or neural disorders. However, in order to obtain the optimal stimulation sequences, it is necessary to develop neural control methods, for example by constructing an inverse model of the target system. For real brains, this can be very challenging, and often unfeasible, as it requires repeatedly stimulating the neural system to obtain enough probing data, and depends on an unwarranted assumption of stationarity. By contrast, detailed brain simulations may provide an alternative testbed for understanding the interactions between ongoing neural activity and external stimulation. Unlike real brains, the artificial system can be probed extensively and precisely, and detailed output information is readily available. Here we employed a spiking network model of sensorimotor cortex trained to drive a realistic virtual musculoskeletal arm to reach a target. The network was then perturbed, in order to simulate a lesion, by either silencing neurons or removing synaptic connections. All lesions led to significant behvaioral impairments during the reaching task. The remaining cells were then systematically probed with a set of single and multiple-cell stimulations, and results were used to build an inverse model of the neural system. The inverse model was constructed using a kernel adaptive filtering method, and was used to predict the neural stimulation pattern required to recover the pre-lesion neural activity. Applying the derived neurostimulation to the lesioned network improved the reaching behavior performance. This work proposes a novel neurocontrol method, and provides theoretical groundwork on the use biomimetic brain models to develop and evaluate neurocontrollers that restore the function of damaged brain regions and the corresponding motor behaviors.

## 1. Introduction

Recent years have seen extraordinary progress in technologies that not only allow us to read the brain signals, but also to artificially stimulate neural circuits. Neurostimulation will be the best way to demonstrate our understanding of neural codes (Stanley, 2013), by injecting signals that produce specific natural sensory sensations (O'Doherty et al., 2011; Choi et al., 2012; Klaes et al., 2014), motor behaviors (Overduin et al., 2012; Van Acker et al., 2013), or memory (Hampson et al., 2013) and cognitive (Hampson et al., 2012) influences. Clinically, a major challenge for the next generation of motor-function-restoring brain-machine interfaces (BMIs) is the incorporation of realistic somatosensory feedback via cortical stimulation (Bensmaia and Miller, 2014). These new research directions will enable neurostimulation tools that provide ongoing dynamic neural modulation to treat brain disorders (Underwood, 2013) and repair brain lesions (Sanchez et al., 2012). These could also potentially be used to induce long-term plasticity leading to lasting recovery (Jackson et al., 2006; Koralek et al., 2012; Song et al., 2013).

Meeting these challenges raises a number of theoretical and technological obstacles. To start with, both neural recording and stimulation are still very limited in terms of scale (number of simultaneous neurons), and of spatial and temporal precision. However, new emerging tools, particularly optogenetics, may provide more precise stimulation of groups of single cells (Suter et al., 2014; Warden et al., 2014). A second obstacle is our still rudimentary understanding of neural coding and brain dynamics, which makes it difficult to provide neurostimulation in a way that is physiologically meaningful. For example, we have only described a small fraction of the large number of cell types and physiological responses in the brain (Douglas and Martin, 2012; Harris and Shepherd, 2015). Understanding the relation between neurophysiology and behavior will require characterizing the interactions between the multiple spatial and temporal scales of the brain, ranging from the molecular (< 1 um and < 1 us) to the macroscopic level (>1 cm and >1 s). Many of these, such as the effect of spatiotemporal input patterns in the dendritic trees or the role of physiological oscillations, remain open questions. Another fundamental component to determine is the high-level algorithms or computations the brain employs to encode and manipulate information (Carandini, 2012). An additional complication is that the brain is a highly non-stationary system, as a result of noisy sensory inputs (even in a highly controlled environment) and internal recurrent dynamics, including modulation by thought, attention, motivation, fatigue, hormones, etc. These factors limit the ability to predict the outcome of neurostimulation and, therefore, to achieve targeted neural control. Consequently, neurostimulation studies have been predominantly confined to measuring the elicited neural responses (Clark et al., 2011; Van Acker et al., 2013).

A biomimetic brain simulation does not suffer from many of these limitations, as it provides a fully reproducible and controllable system with full access to all neurons and synapses. The biomimetic system can also be manipulated to reflect different conditions, such as normal physiology vs. pathophysiology. Models are however limited by their dissimilarity to the brain, which means the solutions found using the model may not be directly applicable to the real brain. In the Discussion we examine the current limitations of our model and what is required to gradually bridge the distance to real applications. Nonetheless, our work can serve as theoretical groundwork toward employing brain simulations to develop and evaluate neural control methods. As models continue to augment their level of detail and realism (Markram et al., 2015), they will provide an increasingly useful tool to help understand the interactions between neurostimulation and ongoing intrinsic neural activity. Previously, we employed biomimetic models to explore the effects of neurostimulation on information flow in neocortex (Kerr et al., 2012), plasticity in somatosensory cortex (Song et al., 2013), and oscillations in primary motor cortex (Chadderdon et al., 2014).

In this paper, we utilize a biomimetic spiking model of sensorimotor cortex connected to a realistic virtual musculoskeletal arm (Dura-Bernal et al., 2015b), which provides a direct link between neural activity and motor behavior. We employ this system to demonstrate the use of a neural control method, based on an adaptive inverse model, to restore behavioral performance of the virtual arm after lesioning of the spiking model. The approach consists of first representing the nonlinear spiking dynamics in a reproducing kernel Hilbert space (RKHS), to enable the use of kernel adaptive filtering techniques (Chen et al., 2012; Li et al., 2013) to construct an inverse model of the sensorimotor spiking network (Li et al., 2015). Then, this inverse model is used to derive neurostimulation patterns that restore the pre-lesion patterns of network spiking activity and thereby partially “heal” or compensate for the lesion. Our results suggest employing biomimetic brain and musculoskeletal models could be useful to study the effects of neurostimulation; and demonstrate the efficacy of the kernel adaptive inverse neurocontroller to repair lesioned neural circuits and restore behavioral performance in the simulation.

## 2. Methods

### 2.1. Sensorimotor system model

**Figure 1:**
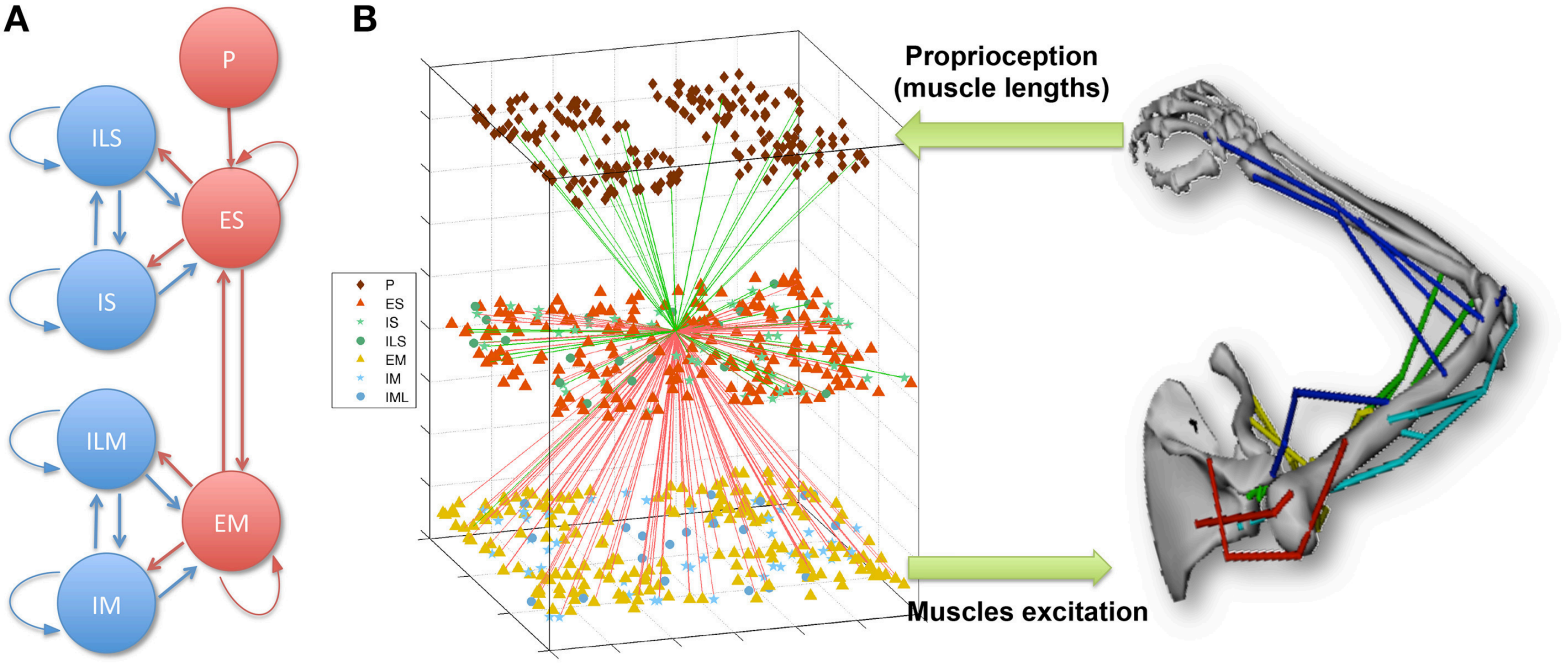
**(A)** Spiking network population connectivity diagram. Proprioceptive (P) units provide input to the sensory (S) population, which is recurrently connected to the excitatory motor (M) population. Both S and M are composed of recurrently connected excitatory (E), fast-spiking (I) and low-threshold spiking (IL) subpopulations. **(B)** Overview of the sensorimotor system interfacing the spiking network with the virtual musculoskeletal arm. The virtual arm receives neural excitation from the EM population and feeds back proprioceptive information to the P population. As an example of cell connectivity, all incoming (green) and outgoing (red) connections of a single ES neuron are shown.

The model of the sensorimotor system consists of a spiking neuronal network connected to a virtual musculoskeletal arm (Figure 1; Neymotin et al., 2013; Dura-Bernal et al., 2015b). The network includes 704 model neurons of 4 different types distributed in 7 subpopulations, and includes cortical-like recurrent connectivity. Using reinforcement learning combined with spike-timing dependent plasticity (STDP), the network is able to learn to drive the virtual arm to reach a target. This section describes the spiking network and musculoskeletal arm models in more detail. The full neuronal network and virtual arm models can be downloaded from ModelDB at http://modeldb.yale.edu/183014.

#### 2.1.1. Spiking neuronal network

Each individual neuron was modeled as event-driven unit which followed a set of rules in order to replicate a set of realistic neuronal features, such as bursting, adaptation, depolarization blockade, and voltage-sensitive NMDA conductance (Lytton and Stewart, 2006; Lytton et al., 2008a,b; Neymotin et al., 2011). This model provides a good trade-off between cell realism and speed of computation, and makes it adequate for simulating networks that include hundreds of these neurons. Each cell had a membrane voltage state variable, which was updated based on three possible events: synaptic input, threshold spike generation, and refractory period. Three types of synaptic inputs were modeled (AMPA, NMDA, and GABA_*A*_), using reversal potentials, time constants and delays consistent with physiological data. Spikes were generated when a voltage threshold was crossed, and were propagated to target neurons after a synaptic conduction delay. After spiking, a relative refractory period was simulated by increasing the threshold potential and adding hyperpolarization. In addition to spikes generated by the network neurons, subthreshold Poisson-distributed spike inputs to synapses were used to provide background activity, representing neural input from surrounding regions not explicitly modeled. A comprehensive description of the cell model equations and parameter values is available in previously published papers (Neymotin et al., 2013; Dura-Bernal et al., 2015b) and on ModelDB.

Input from the virtual arm to the neural network was provided by the proprioceptive (P) population, which consisted of 192 NetStims (NEURON spike generators) and encoded the muscle lengths. P units were divided into four subpopulations, each responsible for representing the mean length of one of four muscle groups: shoulder extensors, shoulder flexors, elbow extensors and elbow flexors. Units employed population coding to represent the muscle lengths, such that within each subpopulation, individual units only fired to a small range of lengths.

The neural network represented a simplified model of the two main elements involved in the sensorimotor cortex learning loop (Wolpert et al., 2011): sensory input and motor output. The sensory (S) and motor (M) populations were each comprised of 192 excitatory cells (ES and EM), 44 fast-spiking inhibitory cells (IS and IM), and 20 low-threshold inhibitory cells (ILS and ILM). Recurrent connectivity was present within each cell class, between the excitatory and inhibitory neurons of each population, and between the two main excitatory cell classes (ES and EM) (Figure 1). Cells were connected randomly based on a probability of connection and weights that depended on the presynaptic and postsynaptic cell class and location. ES cells received input from the P units encoding muscle lengths, which enabled them to represent the arm posture by combining information from multiple muscle lengths. The output of EM neurons, which received strong afferent inputs from the ES cells, was used to generate the muscle excitations sent to the virtual arm. Excitation to each muscle group was calculated by summing the number of spikes of the corresponding EM subpopulation (48 neurons for each muscle group) over an 80 ms sliding window, and threshold-normalizing the value between 0 and 1.

The sensorimotor system therefore forms a closed-loop circuit: virtual arm muscle lengths are encoded in the P population, P units project to the S population, which in turn projects to the M population, which provides excitation to the virtual arm muscles. By employing reinforcement learning—reward or punishment depending on whether the arm is getting closer or farther from the target—to modify the synaptic weights via spike-timing dependent plasticity (STDP), the network can learn to drive the arm to a target. This requires a training phase where exploratory movements are enforced in the virtual arm, in order to explore the space and generate the appropriate mapping between input sensory information and output motor commands (Neymotin et al., 2013; Dura-Bernal et al., 2014, 2015b). Currently, the network can only be trained to reach one target at a time. However, it can be extended to learning multiple targets by adding a population to encode target selection, as we (Dura-Bernal et al., 2015a) and others (Spüler et al., 2015) have shown.

The spiking network simulations were run in NEURON 7.3 (Hines and Carnevale, 2001; Carnevale and Hines, 2006) on a Linux workstation with 24 Intel Xeon 2.7 GHz cores and on a High-Performance Computing system with 512 AMD Opteron 2.6 Ghz cores.

#### 2.1.2. Virtual musculoskeletal model

The virtual arm consists of a biomechanical and dynamical model of the upper right limb musculoskeletal system. The original model (Holzbaur et al., 2005), downloadable from the SimTK website (http://simtk.org/home/up-ext-model), was adapted for our purposes by limiting it to shoulder and elbow joint rotation (two degrees of freedom) in the horizontal plane. The model includes 8 skeletal rigid bodies that serve to anchor 18 different muscles, responsible for shoulder extension and flexion, and elbow extension and flexion.

Virtual arm joint kinematics and dynamics were based on anatomical studies, match experimental measurements of an average-sized human adult male, and were implemented using an extension of the Hill-type muscle model (Zajac et al., 1989; Schutte et al., 1993; Thelen et al., 2003). In this model, muscle forces are parameterized based on the optimal fiber length, peak force, tendon slack length and pennation angle, and calculated as a function of four variables: the muscle and tendon lengths, contraction velocity and muscle fiber activation. In turn, muscle activation is derived from an ordinary differential equation driven by an external signal: the muscle excitation. In our system, muscle excitation is provided by the motor population of the spiking network model. The arm kinematics, including position, velocity and acceleration of each joint, are then computed based on the muscle forces using the recursive Newton-Euler algorithm (Featherstone and Orin, 2000). Further details are available in our previous work (Dura-Bernal et al., 2015b) and via ModelDB.

### 2.2. Adaptive inverse model for neural control

We aim to restore the neural response of the EM population to its pre-lesion state, and consequently recover the original reaching trajectory. A set of probing neurostimulation sequences and their corresponding neural activity were recorded and used to construct an inverse model of the stimulus-response pairs. The goal is to automatically generate a set of optimized neurostimulation patterns given a desired neural response. To learn this inverse mapping, we applied the kernel adaptive filtering (KAF) method (Liu et al., 2010).

#### 2.2.1. Kernel adaptive filtering

Kernel methods (Scholkopf and Smola, 2001) form a powerful unifying framework in classification, clustering and regression, contributing to many impactful applications in machine learning, signal processing and biomedical engineering. KAF is an online learning technique that combines adaptive signal processing with kernel methods. The idea is to transform the input data into a richer and potentially infinite-dimension feature space via a positive definite reproducing kernel, such that the inner product operation in the feature space can be computed efficiently in the input space through the kernel evaluation. KAF performs classical online linear methods in the enriched feature space or reproducing kernel Hilbert space (RKHS). This way it moves beyond the limitations of the linear model to provide general nonlinear solutions in the original input space. This input space is not restricted to numeric data, and could consist, for example, of spike trains (Li et al., 2013). KAF brings together adaptive signal processing and feedforward artificial neural networks, by combining the best of both worlds: the universal approximation property of neural networks and the simple convex optimization of linear adaptive filters.

In the family of kernel adaptive filters, the kernel least mean square (KLMS) algorithm (Liu et al., 2008) is the simplest stochastic gradient descent method that operates on the instantaneous error. A finite impulse response (FIR) filter trained in the RKHS using the least mean squares (LMS) algorithm, it can be viewed as a single-layer feedforward neural network or perceptron. For a set of *n* input-output pairs {(**u**_1_, *y*_1_), (**u**_2_, *y*_2_), …, (**u**_*n*_, *y*_*n*_)}, the input vector $u_{i}\in 𝕌\subseteq {\mathbb{R}}^{m}$ (where 𝕌 is a compact input domain in ℝ^*m*^) is mapped into a potentially infinite-dimensional feature space 𝔽. Define a 𝕌 → 𝔽 mapping φ, the feature-space parametric model becomes

(1) $$\begin{array}{ll} ŷ=\hat{f}\left(u\right)=\Omega^{T}\varphi \left(u\right) & \; \end{array}$$

where Ω is the weight vector in the RKHS. Using the representer theorem (Scholkopf et al., 2001) and the “kernel trick,” (1) can be written as

(2) $$\begin{array}{ll} \hat{f}\left(u\right)=\overset{n}{\underset{i=1}{\sum }}\alpha_{i}K\left(u_{i},u\right) & \; \end{array}$$

where K(**u**, **u**′) is a Mercer kernel, corresponding to the inner product 〈φ(**u**), φ(**u**′)〉, and α_*i*_ are the weight coefficients. The most commonly used kernel is the Gaussian kernel

(3) $$\begin{array}{l} K_{a}(u,u\prime )=\exp\left(-a{\left\|u-u\prime \right\|}^{2}\right) \end{array}$$

where *a* > 0 is the kernel parameter. To effectively address the growth of the radial basis function structure in KAF, the quantized version of the KLMS algorithm was used (Chen et al., 2012).

#### 2.2.2. Reproducing Kernel Hilbert Space (RKHS) for spike trains

Here, we briefly describe the reproducing kernel used to map the neural responses to a RKHS for spike trains. A spike train or sequence of *M* ordered spike times, i.e., ***s*** = {*t*_*m*_ ∈ T:*m* = 1, …, *M*} in the interval T = [0, *T*], can be viewed as a realization of an underlying stochastic point process with conditional intensity function λ(*t*|*H_t_*), where *t* ∈ T = [0, *T*] denotes the time coordinate, and *H_t_* is the history of the process up to *t*. Spike trains can be mapped into an RKHS by defining a strictly positive definite kernel, the Schoenberg kernel, between the conditional intensity functions of two point processes (Paiva et al., 2009) as

(4) $$\begin{array}{ll} K_{a_{\lambda }}\left(\lambda \left(t|H_{t}^{i}\right),\lambda \left(t|H_{t}^{j}\right)\right)= & \;\\ \;\exp\left(-a_{\lambda }\underset{\tau }{\int }{\left(\lambda \left(t|H_{t}^{i}\right)-\lambda \left(t|H_{t}^{j}\right)\right)}^{2}dt\right) & \; \end{array}$$

where *a*_λ_ > 0 is the spike-train kernel parameter. The intensity function can be estimated by convolving the spike times with a smoothing kernel *g*(*t*), yielding

(5) $$\begin{array}{ll} \hat{\lambda }\left(t\right)=\overset{M}{\underset{m=1}{\sum }}g\left(t-t_{m}\right),\left\{t_{m}\in T:m=1,\ldots \;,M\right\}. & \; \end{array}$$

**Figure 2:**
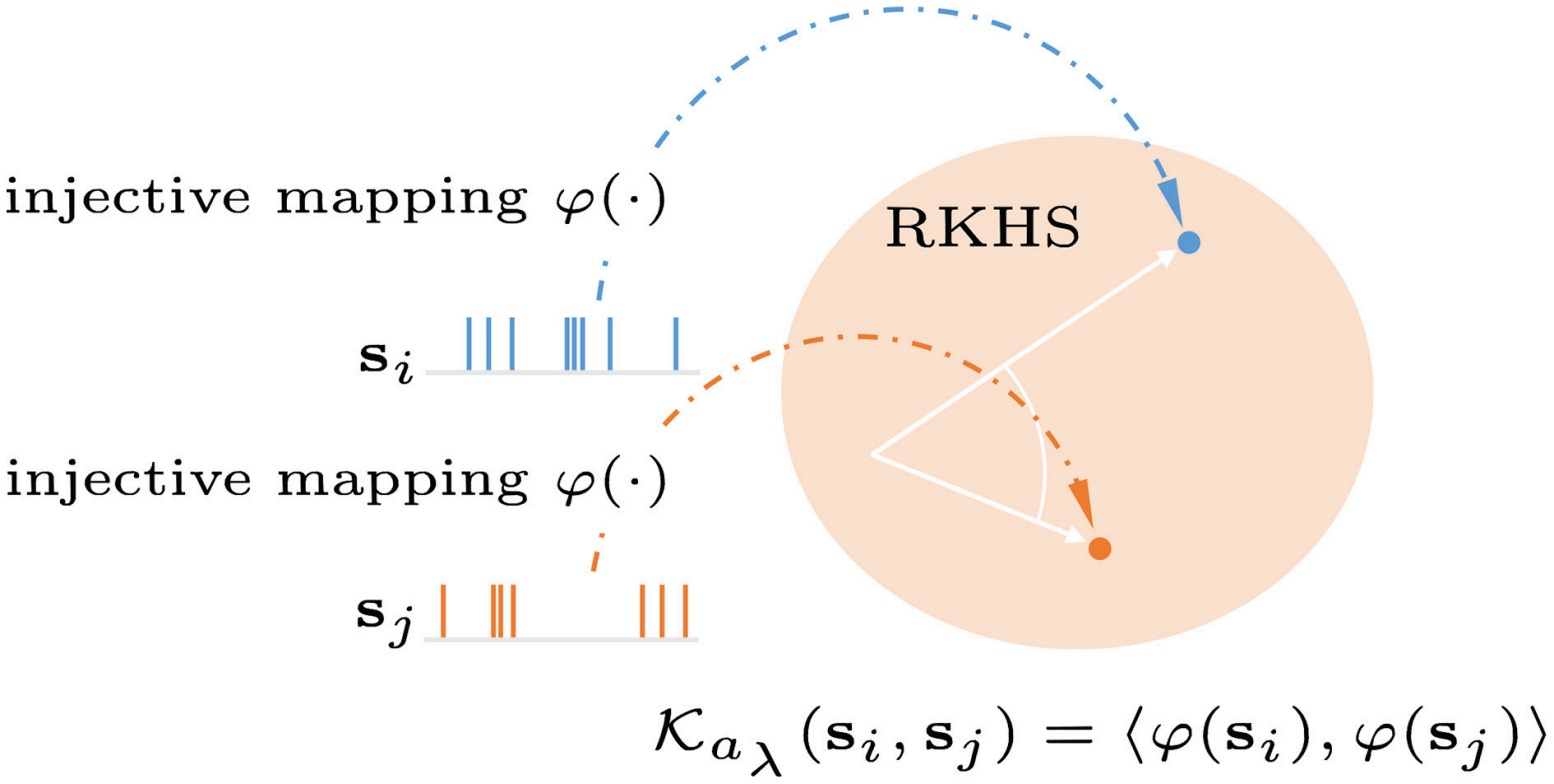
**Graphical representation of a reproducing kernel Hilbert space defined on spike trains using the Schoenberg kernel**. Spike trains are transformed into a high dimensional feature space (Hilbert space). Applying the kernel trick allows inner products in this space to be computed without explicit reference to the feature vectors.

As shown in Figure 2, the entire spike train is mapped into a location or function in the RKHS. For simplicity, we use the rectangular function $g\left(t\right)=\frac{1}{T}\left(U\left(t\right)-U\left(t-T\right)\right)$, where T≫ the inter-spike interval, and *U*(*t*) is a Heaviside function. Let $s_{i}^{n}\left(t\right)$ denote the spike train for the *i*-th sample of the *n*-th spiking unit, the multi-unit spike kernel is taken as the unweighted sum over the kernels on the individual units

(6) $$\begin{array}{ll} K\left(s_{i}\left(t\right),s_{j}\left(t\right)\right)=\underset{n}{\sum }K\left(s_{i}^{n}\left(t\right),s_{j}^{n}\left(t\right)\right). & \; \end{array}$$

#### 2.2.3. Inverse model of spiking network

**Figure 3:**
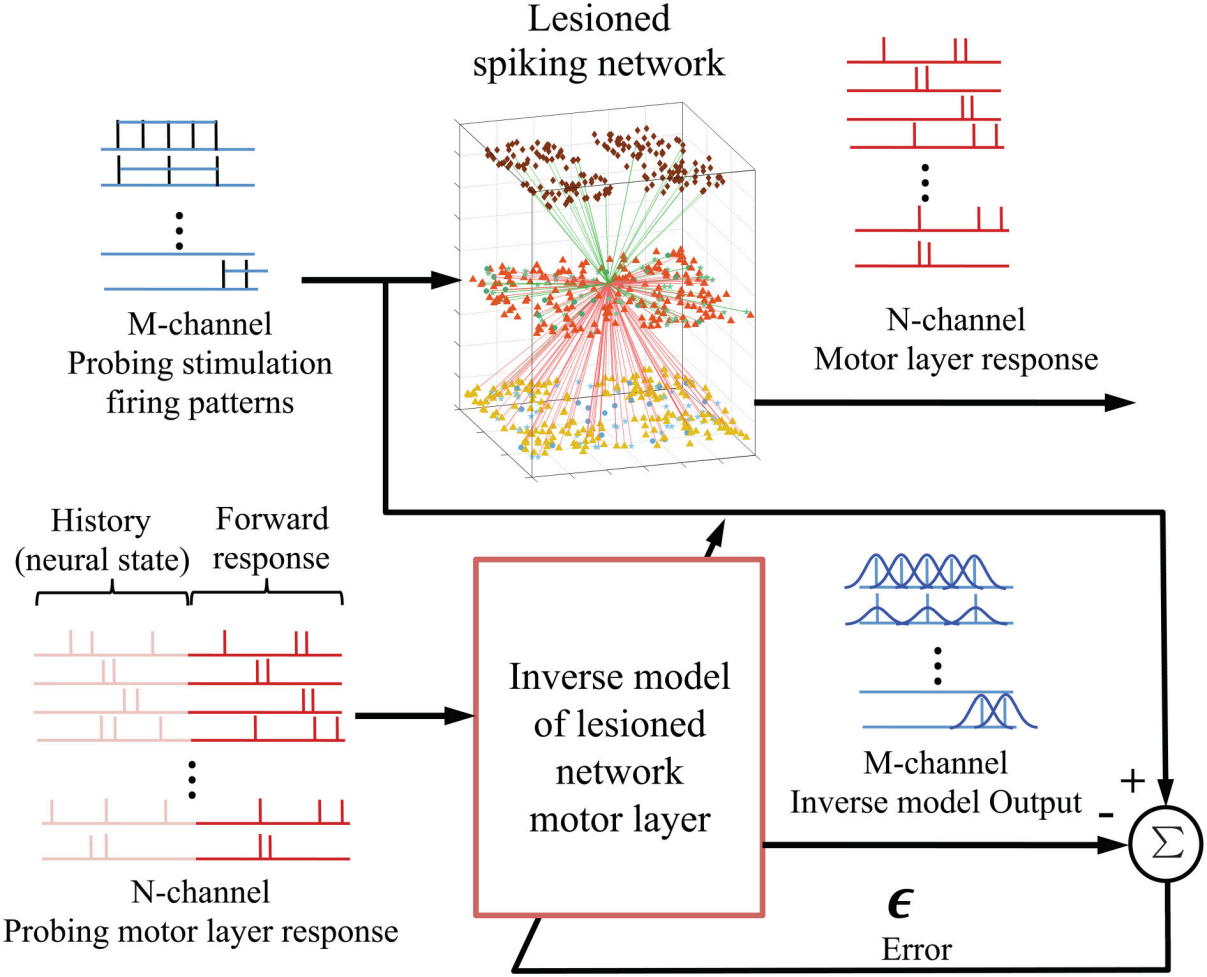
**Integration of kernel adaptive inverse controller with biomimetic spiking model of sensorimotor system**. The M-channel probing neurostimulation firing pattern (rates) and the corresponding N-channel EM population responses are used to train an inverse model or mapping of the lesioned spiking network motor layer. The motor layer response window includes the history (provides current state) and the forward response. Once fully trained, the inverse model outputs an estimate of the optimal neurostimulation for a desired neural response.

As shown in Figure 3, the goal is to learn an inverse model of the plant ***P***, i.e., lesioned spiking network motor layer, from the probing stimulus-response pairs; and then apply the pre-lesioned motor response to the trained multiple-input-multiple-output (MIMO) decoding model to obtain an optimized repair neurostimulation pattern. The neural responses of the EM population were mapped into a RKHS for spike trains and the filter coefficients were adapted using errors between the desired probing neurostimulation firing rates and the inverse model output. Using cross validation, the kernel parameter in (4) was set at *a*_λ_ = 0.05. The allowable neurostimulation duration was fixed at 200 ms, from the 100 to 300 ms mark of each trial.

**Figure 4:**
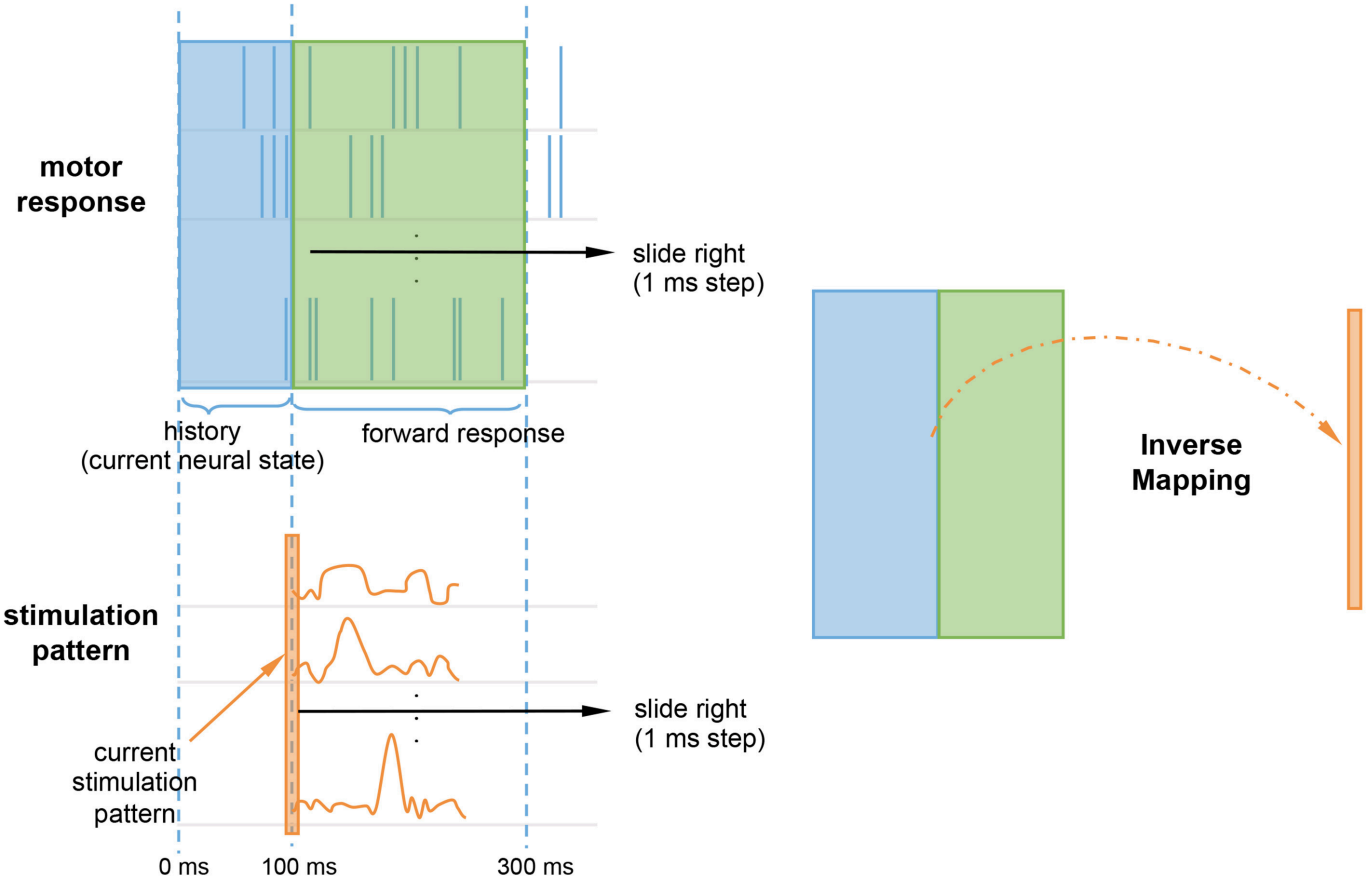
**Inverse mapping of motor response onto the stimulation pattern**. At every time step, a sliding window containing the motor forward response (green) and history (blue) is mapped to the current stimulation pattern (orange). The history accounts for non-stationarity by providing the system with the current neural state. The current stimulation pattern consists of a vector of firing rates for the current time step.

Rather than continuously stimulating the spiking network, we were interested in whether an initial onset of neurostimulation patterns could repair the lesioned reaching trajectories. The topological relationship between the probing stimuli and responses was set using a delay embedding of 300 or 200 ms, for the left and bottom target, respectively. The delays were selected to reflect the expected duration of the stimulation effect or impulse response for each target, determined by subtracting the end the stimulation period (300 ms) from the reaching trajectory duration. To account for the non-stationarity of the spiking network, the stimuli was not only mapped to the forward response, but also to the history of the motor response (Figure 4). The rationale being that, like state machines, to calculate the correct next state, the system needs to know both the input and the the current neural state. The history, or backward motor response, provides the current neural state. Therefore, every 1 ms step, a sliding window containing both the history and the forward response is mapped to the current stimulation pattern. The current stimulation pattern consists of the vector of *m* firing rates for that time step, where *m* is the size of the ES population. Since stimulation starts at 100 ms, the initial history window size only ranges 0–100 ms, but will grow to match the size of the forward response window as time progresses.

The stimulation patterns generated by the inverse model were input in the spiking network by connecting spike generators (NetStims) to each of the ES population cells. Different stimulation patterns were characterized in terms of the targeted cell, starting time, duration, and firing rate of the external spike generator, which provided input via the AMPA receptor synapse of the cell. The synaptic weight between the spike generator and the cell was ten times larger than that of regular background synaptic inputs.

The Matlab code for the inverse neurocontroller, the probing dataset, and the Python/NEURON code to lesion and stimulate the spiking network are available online at: https://github.com/Neurosim-lab/controller_spiking_network_arm.

## 3. Results

Initial trained reaching trajectories were perturbed by different types of simulated lesions of the sensorimotor network. These lesions were then repaired using the adaptive inverse controller which aimed to restore the spiking patterns and reaching behavior. Repair commenced by generating a set of probes for the perturbed system, in order to inform the inverse model.

### 3.1. Simulated lesions and response probing data

**Figure 5:**
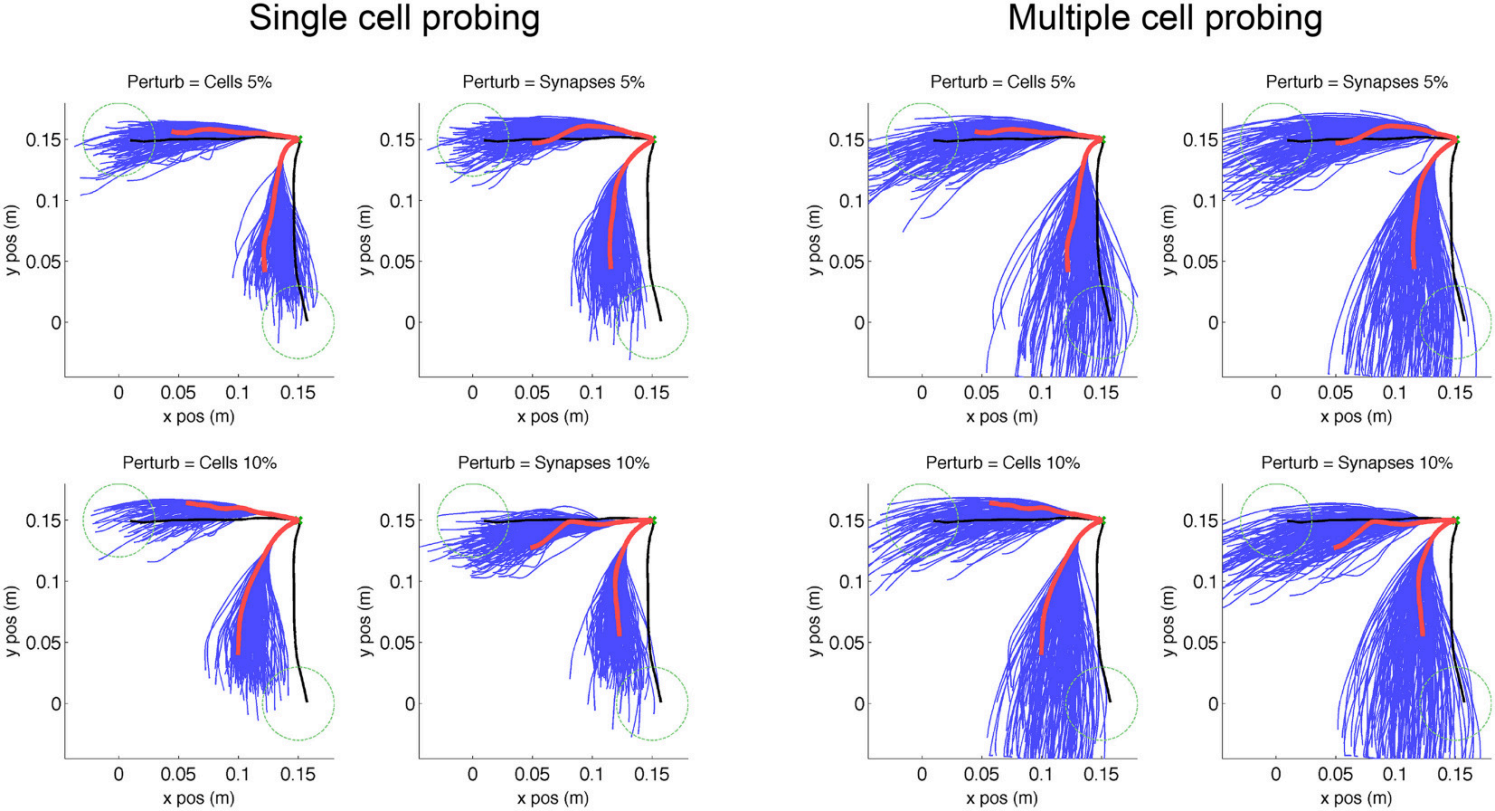
**Virtual arm trajectories after simulated lesion and neurostimulation probing**. Original arm trajectories (black), were perturbed due to a network lesion leading to decreased reaching performance (red). A set of single cell and multiple cell probing stimulations (blue) enabled the neurocontroller to build an inverse model of the system. Data is shown for 2 targets and 4 types of perturbation. Target is indicated as green dashed circle.

Original trajectories were obtained from the spiking network, after training it via reinforcement learning STDP to drive the virtual arm to a specified target. The setup was similar to that of a common sensorimotor experimental task, the center-out reaching task (Hatsopoulos et al., 2004; Hwang and Shadmehr, 2005; Sanchez et al., 2011). The subject starts from a central position in a horizontal 2D plane and has to reach one of the targets in a surrounding circumference. For the purpose of evaluating the neurocontroller, we chose two different targets (bottom and left) located 15 cm from the starting position. These targets were chosen because they involve different sets of muscles: shoulder extensor and elbow flexor for bottom target; and shoulder flexor and elbow extensor for left target (Figure 5). Reaching duration was 600 ms for the left target and 500 ms for the bottom target. Target distance as well reaching durations (or velocities) are consistent with experimental data of humans and primates performing a similar task (Hatsopoulos et al., 2004; Hwang and Shadmehr, 2005; Sanchez et al., 2011).

We simulated two types of lesion for each of the trained targets. The first type of lesion was cell ablation, which consisted of removing 5% or 10% of cells randomly selected from the 192 excitatory-sensory (ES) cells. These cells projected to the excitatory-motor (EM) cells which were the eventual target of trajectory repair. The second type of lesion was removal of 5% or 10% of synaptic connections, selected randomly from the all 21,588 connections in the network. Behavioral degradation was significant for both types of lesions, as seen by the difference between original (Figure 5, black) and post-lesion trajectory (Figure 5, red).

The kernel-based inverse model of the neural system was generated by probing the response of the system to different neurostimulation patterns. Each of the 8 scenarios (2 targets × 2 lesion types × 2 percentages) was probed by stimulating each (remaining) individual ES cells for 200 ms with input spike rate of 250 Hz via AMPA receptors, starting 100 ms after reaching trial onset. A second probing dataset consisted of randomized multiple-cell stimulation patterns to the ES population (1–10 cells), starting at 100 ms and with a random duration between 100 and 500 ms (Figure 5, blue lines). These trajectories reflect the effect of the lesion plus the probing neurostimulation.

### 3.2. Restoration of pre-lesion spiking patterns and trajectories via neurostimulation

The neurostimulation patterns required to reproduce desired output population neural patterns were obtained using the inverse model generated from the probing data input/output correspondences. This inverse solution determines the pattern of activation of ES cells (inputs) that will be needed in order to get a particular desired output pattern from the EM cells (outputs). The EM cells then provide motor commands to the virtual arm to produce the trajectory. It is desirable to produce the repairing neurostimulation patterns in the early stages of the reaching task, in order to correct the trajectories before they have strayed too far from the desired direction.

**Figure 6:**
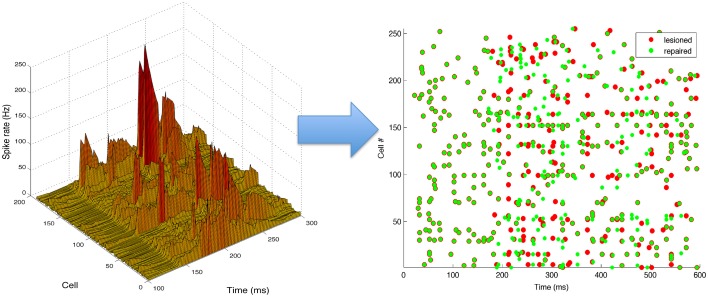
**Neurostimulation pattern derived by the kernel adaptive inverse neurocontroller for the 10% cell lesion of left target reaching**. (Left) The pattern is represented in a continuous three-dimensional space that includes the cell number, time and intensity (rate). (Right) Raster plot of activity before (red) and after neurostimulation (green), illustrating the spiking changes derived from neurostimulation (e.g., increased rate of cell 170, from 200 to 300 ms). All S population cells (1–255) are shown, including inhibitory ones (192–255), although only the excitatory cells (1–191) can be stimulated.

An example of a neurostimulation pattern derived by the inverse model is illustrated in Figure 6 using a 3D representation of the parameter space explored by the algorithm, with dimensions corresponding to the cell number, time and intensity (firing rate) of the neurostimulation. The left panel shows a complex spatiotemporal pattern with relatively strong inputs to 12 ES neurons, with the highest stimulation targeting cell 170 at ~200 ms. Neurostimulation is reflected in the ES cells raster plot (right panel) as a set of new spikes (e.g., cell 170 between 200 and 300 ms). These stimulations then shift activations more broadly due to divergent multisynaptic effects, resulting in changes to the target EM population. Note that the effect of neurostimulation on the ES cells will depend on a number of interacting factors, including co-occurring excitatory and inhibitory inputs from other cells, or the intrinsic state of the cell (e.g., hyperpolarized).

**Figure 7:**
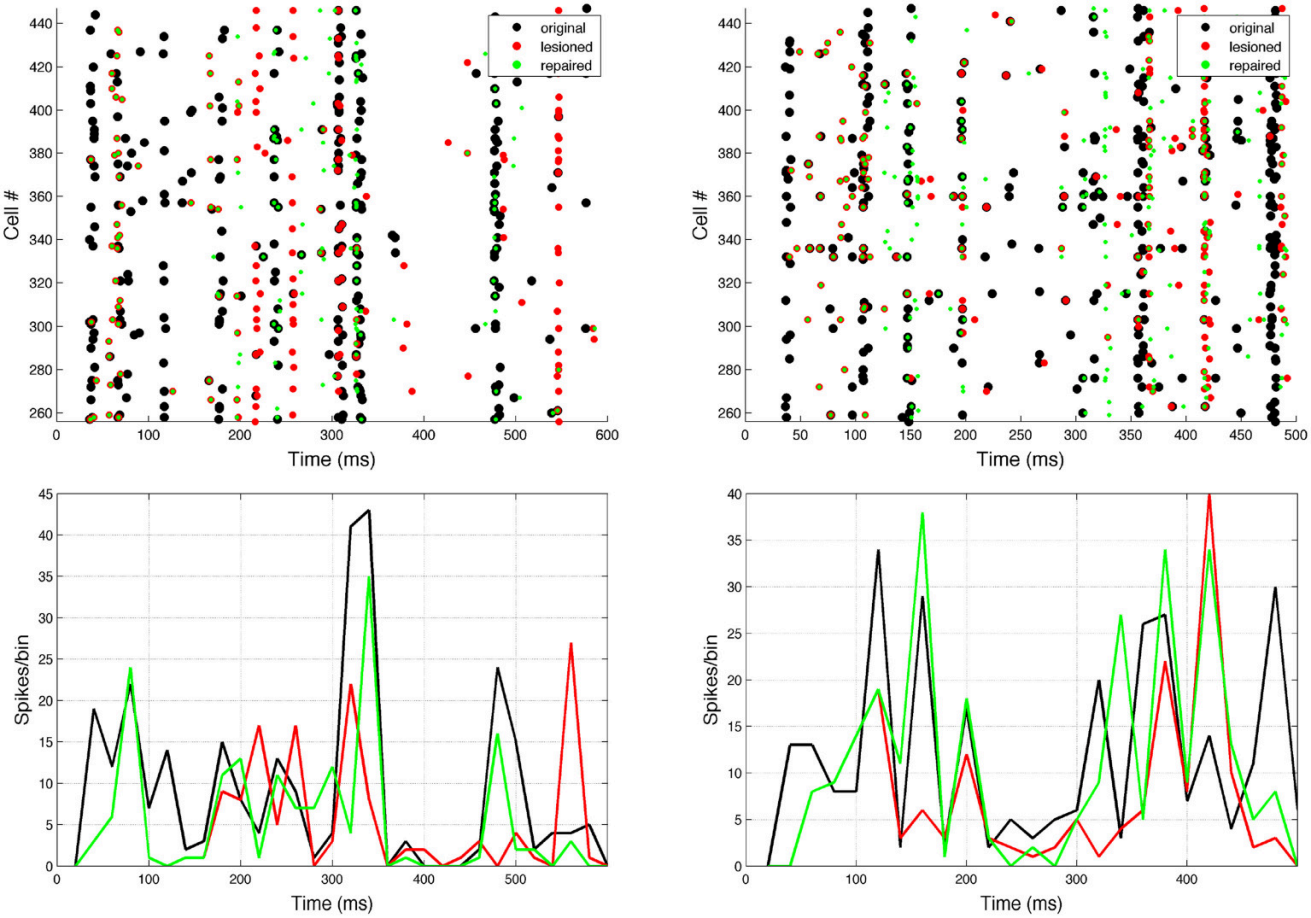
**Raster plot and population peri-event time histogram (PETH) of the original (black), lesioned (red), and repaired (green) motor population**. Overlaps of black and green dots or lines indicate activity that has been restored via neural stimulation. Note how before the neurostimulation is delivered, the activity of the lesioned and repaired networks is identical. Two representative examples are shown: 10% cell lesion of left target reaching (left panel), and 10% synaptic lesion of bottom target reaching (right panel). Cells ids correspond to the motor population (256–511). PETH bin size is 20 ms.

Neurostimulation administered to ES cells recovered some of the original EM spiking patterns (Figure 7). Comparing lesioned (red) with control (black) activity demonstrates that the lesion had a major impact on activity patterns, due to the high recurrent connectivity of the network. Neurostimulation then brought the activity back closer to the original (compare green to black), including restoration of coordinated bursts of activity with precise timing. Two representative examples are included to illustrate the variants in terms of firing patterns for different targets and types of lesions.

We employed three different spike-train metrics and two behavioral metrics to evaluate the performance of our system. The *SPIKE-distance* metric (Kreuz et al., 2011, 2015) was used to calculate pairwise dissimilarities between all cells in the motor population (0–1 measure, 0 means identical spike trains), and averaged across all cells. Similarly, the *SPIKE-sync* metric (Kreuz et al., 2011, 2015) quantified the degree of synchrony between pairs of cells. This metric can be understood as a coincidence detector where the coincidence window is defined adaptively according to the local firing rate. The third metric was the correlation coefficient between the peri-event stimulus histograms (PETHs). To avoid biases due to initial phase, we averaged the result across *N* − 1 histograms with increasing starting times (1ms steps), where *N* is the PETH bin size (20 ms). In terms of behavioral metrics we employed the final distance to target at the end of the reaching trial, and the mean point-wise distance along the trajectory.

**Figure 8:**
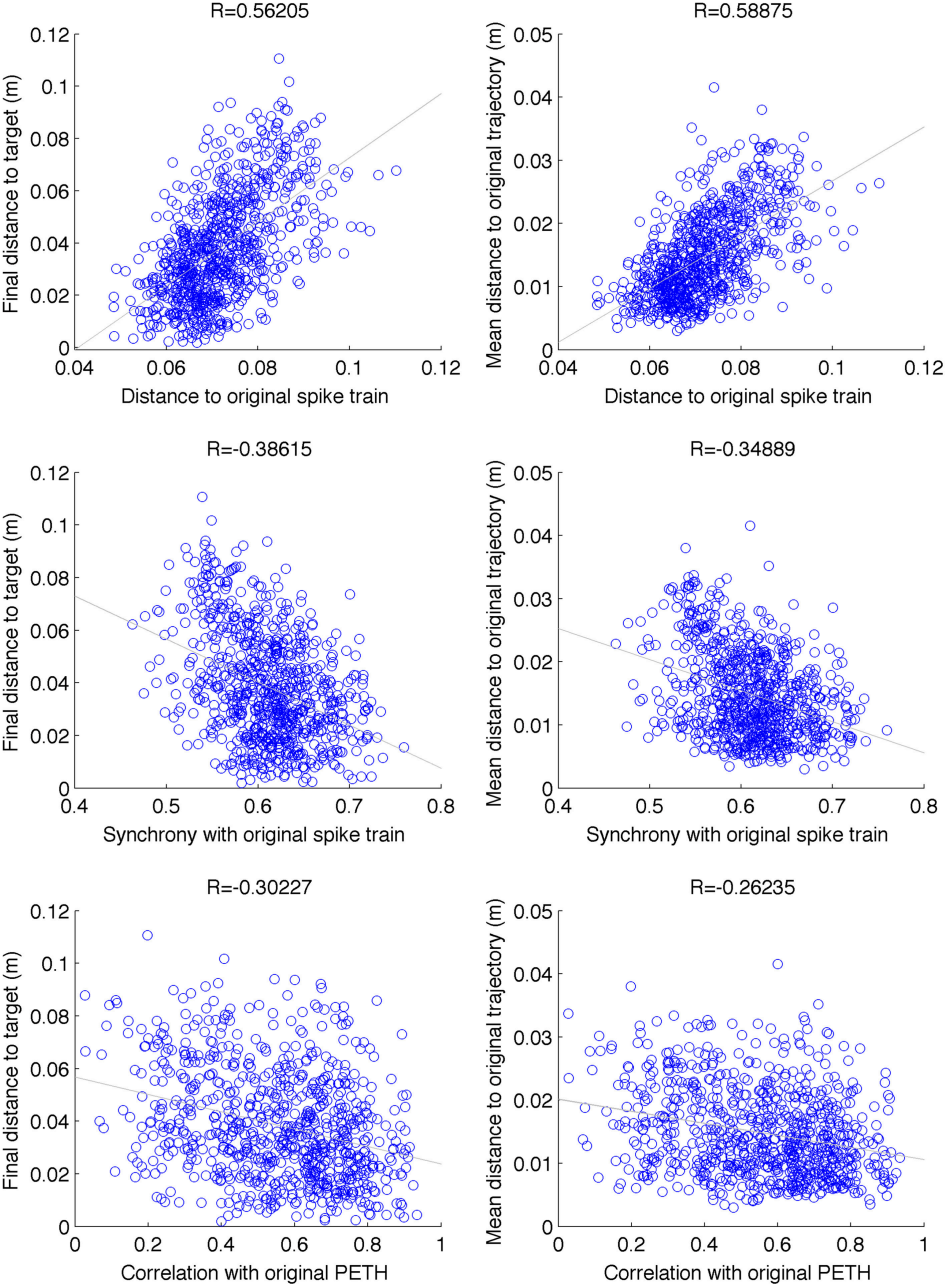
**Scatter plot between different spike-train distance metrics (between original and perturbed) and behavioral performance**. Data is shown for 100 random perturbations for each of the 2 targets × 2 types (cell vs synapse) × 2 intensities (5% and 10%) (*N* = 800). Final distance to target and mean distance to original trajectory were positively correlated with distance to original spike-train (SPIKE-distance metric), and negatively correlated with the level of synchrony with respect to the original spike-train (SPIKE-sync metric) and the correlation with the original PETH. These relations can be used as a reference to evaluate the neurostimulation results. *p* < 0.001 for all correlations.

By analysing 800 random perturbations of the original trajectories, equally distributed among the 2 targets and 4 perturbation types, we found a a relatively strong and significant correlation (|*R*| = 0.41, *N* = 800, *p* < 0.001) between the spike train and behavioral metrics (Figure 8). The strongest correlation was found between the distance to original spike train (SPIKE-distance) and behavioral metrics (*R* = 0.57). These relations provide a useful reference system to evaluate the results of the repair neurostimulations. At the same time they highlight one of the challenges of this approach: the system includes multiple stages of complex nonlinear dynamics, ranging from the single cell to the virtual arm model. As a consequence, similarity to original spike train is not sufficient to guarantee recovery of the original arm trajectory.

**Figure 9:**
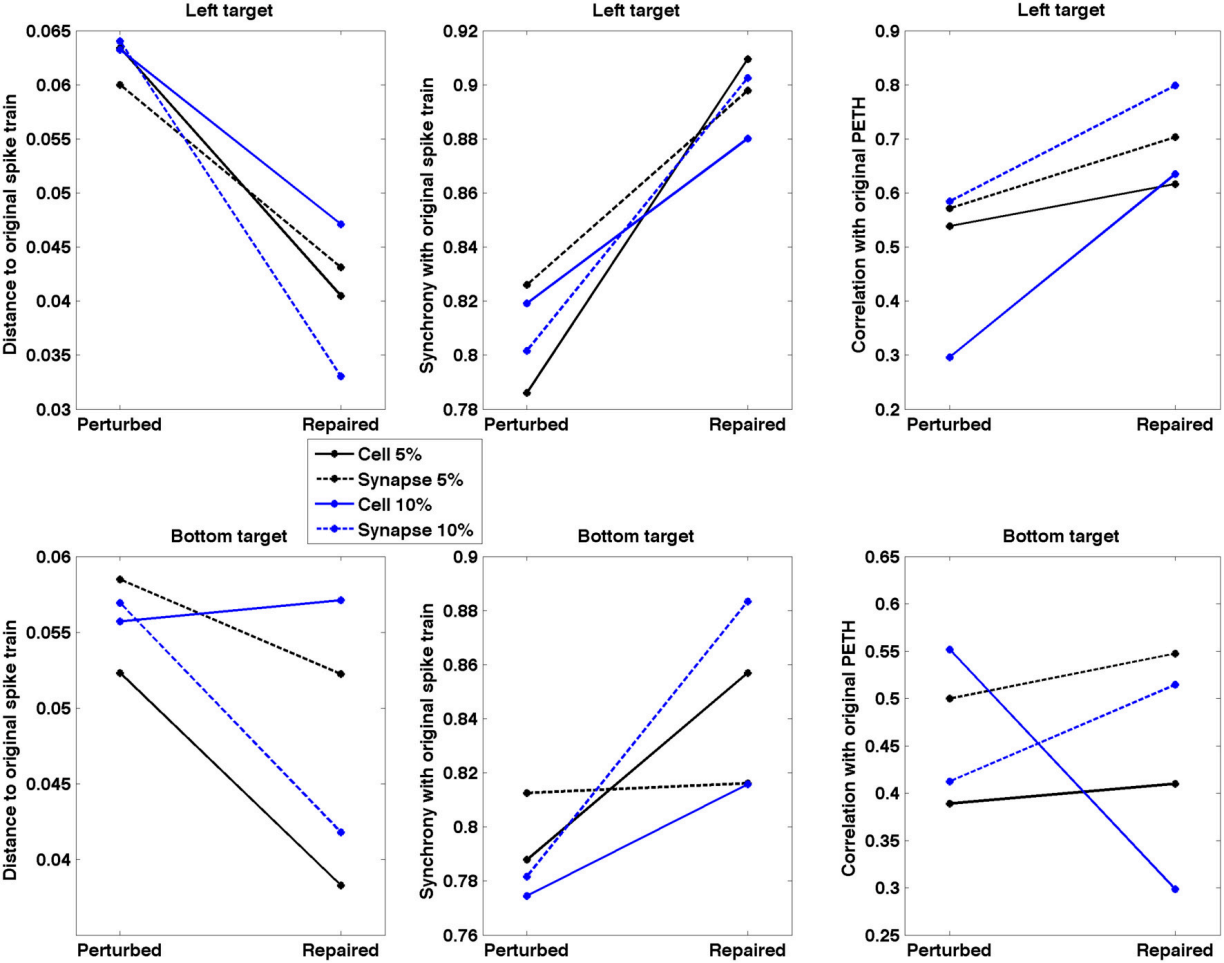
**Spike train dissimilarity (SPIKE-dist), spike train level of synchrony (SPIKE-sync) and population PETH correlation between original and lesioned/repaired networks**. Data shown for two different targets and four lesion types. Neurostimulation reduced spiking pattern dissimilarity and increased synchrony between the lesioned and original networks for 7/8 conditions; and increased PETH correlation for 6/8 conditions.

Repaired spike patterns were overall closer to the original ones than the lesioned patterns (Figure 9). All except one (7/8) conditions exhibited a decrease in spike train dissimilarity (SPIKE-dist, mean decrease = 0.017) after neurostimulation. Neurostimulation also increased spike train synchronization with respect to the original pattern (SPIKE-sync, mean increase = 0.072) in all conditions. The correlation between the original and repaired population PETH (20 ms bins) was higher than between the original and perturbed PETH for 7/8 conditions (mean increase = 0.133). Repair of the bottom target 10% cell lesion resulted in an improvement of the synchronization measure, but a decline in the spike distance and PETH correlation measures. This evidences the difficulty and ambiguity that arise when comparing firing patterns, and underlines the need to identify what features of the spike trains are more relevant to the task, as well as to complement the system evaluation with direct behavioral metrics.

**Figure 10:**
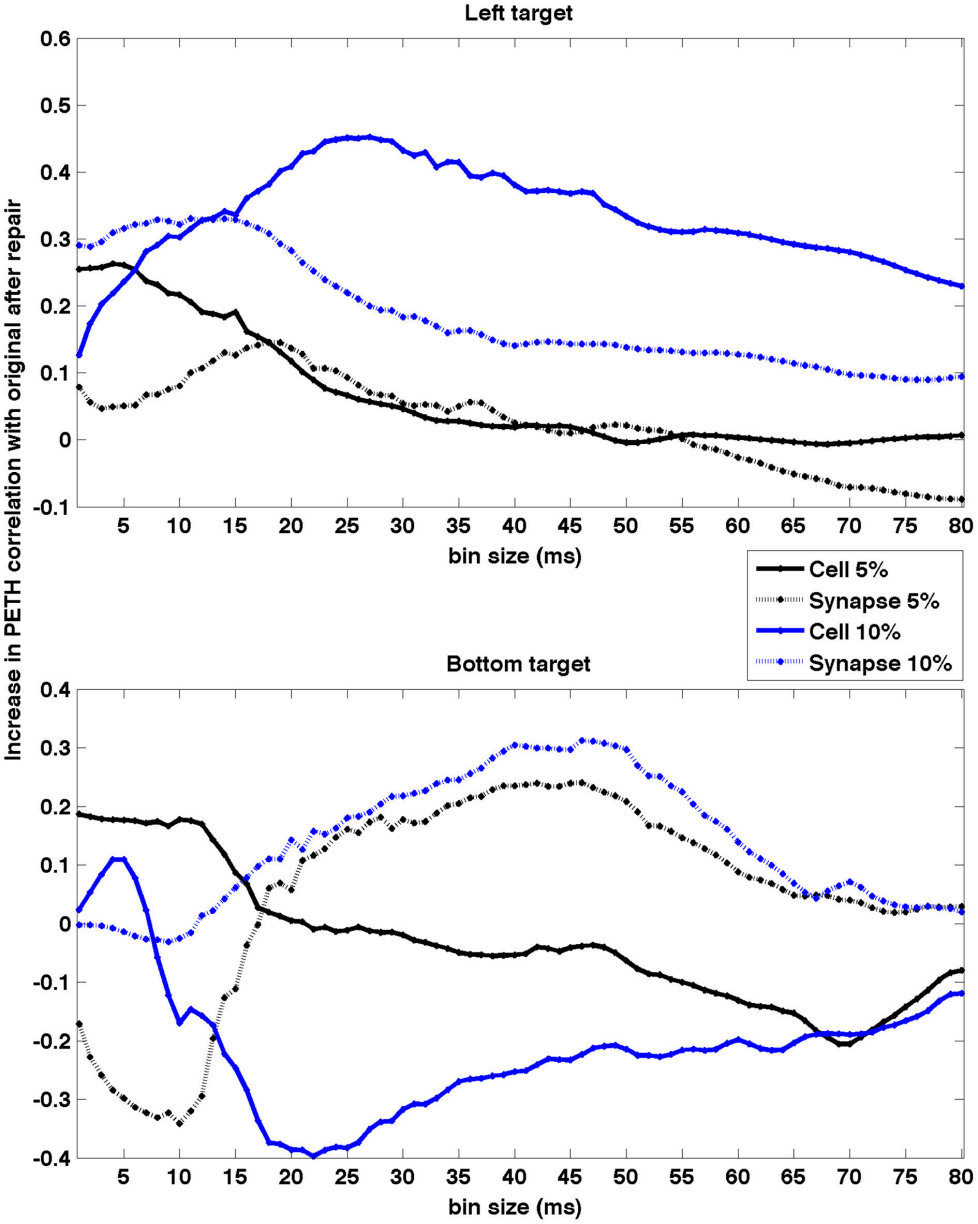
**Effect of PETH bin size on PETH correlation between original and repaired vs. lesioned**. Data shown for two different targets and four lesion types. The effect was dependent on type of lesion and target. Higher restoration for smaller bin sizes (< 20 ms) suggests spike synchrony may play a dominant role, whereas for larger sizes (~ 30–50 ms) suggests firing rate is the predominant factor. PETHs were calculated using firing rate to enable comparison of different bin sizes.

To quantify how the timescale of the analysis affected the neurocontroller results, we evaluated the effect of PETH bin size on the population PETH correlation increase after neurostimulation (Figure 10). This can potentially provide insights into what timescales are more relevant for restoration. Our results indicate different trends depending on the lesion and target. Five of the conditions exhibit higher restoration for smaller bin sizes (< 20 ms) which suggests that spike synchrony played a dominant role. This contrasts with the remaining three conditions where the top restoration performance was obtained for larger bin sizes (~30–50 ms), suggesting firing rates were the predominant factor.

**Figure 11:**
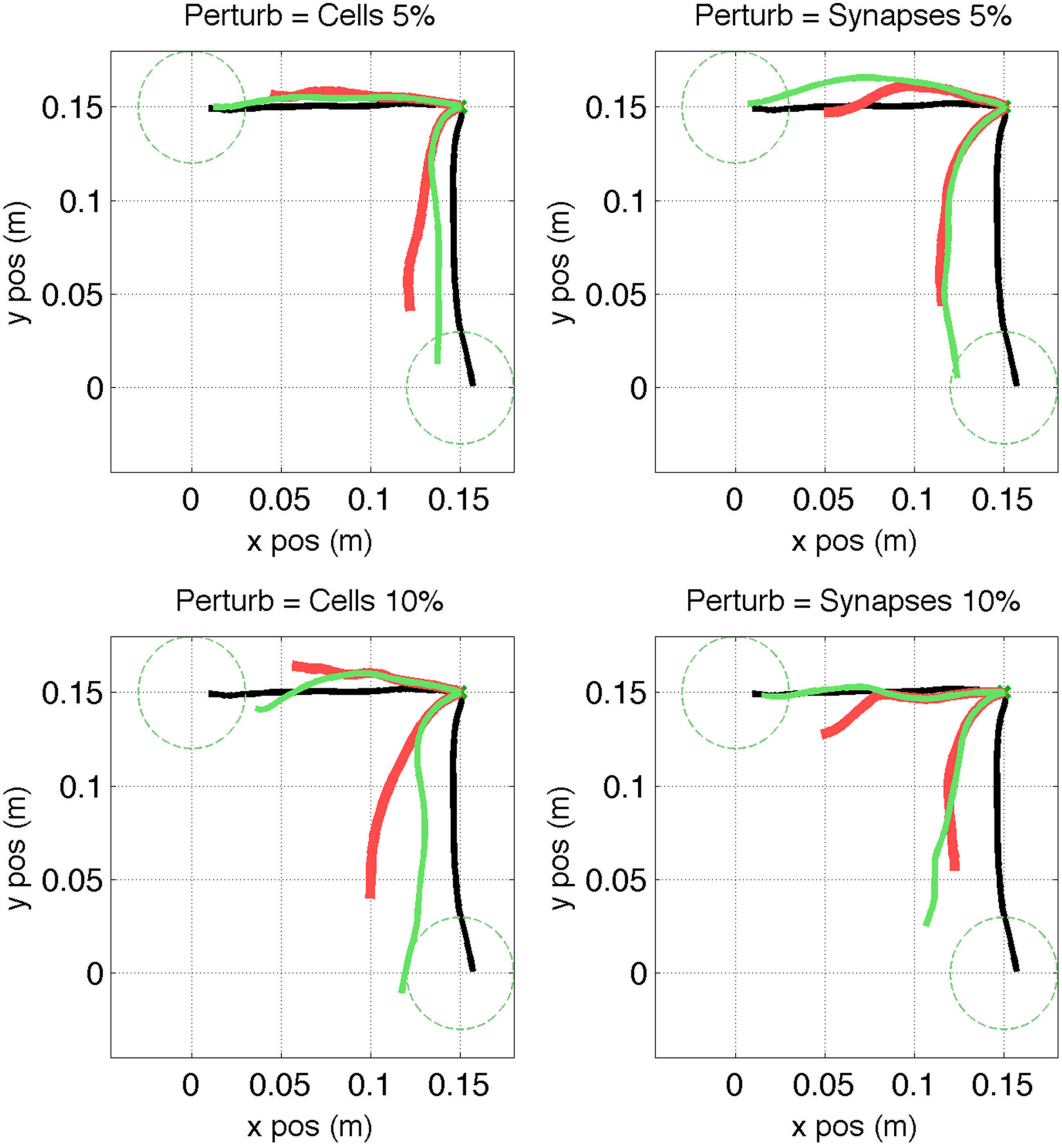
**Virtual arm trajectories after simulated lesion and repair neurostimulation**. Original arm trajectories (black), were perturbed due to a network lesion leading to decreased reaching performance (red). Neurostimulation partly repaired the network and restored the target reaching performance (green). Results are shown for 2 targets and 4 types of perturbation. Target area is indicated as dashed circle.

**Figure 12:**
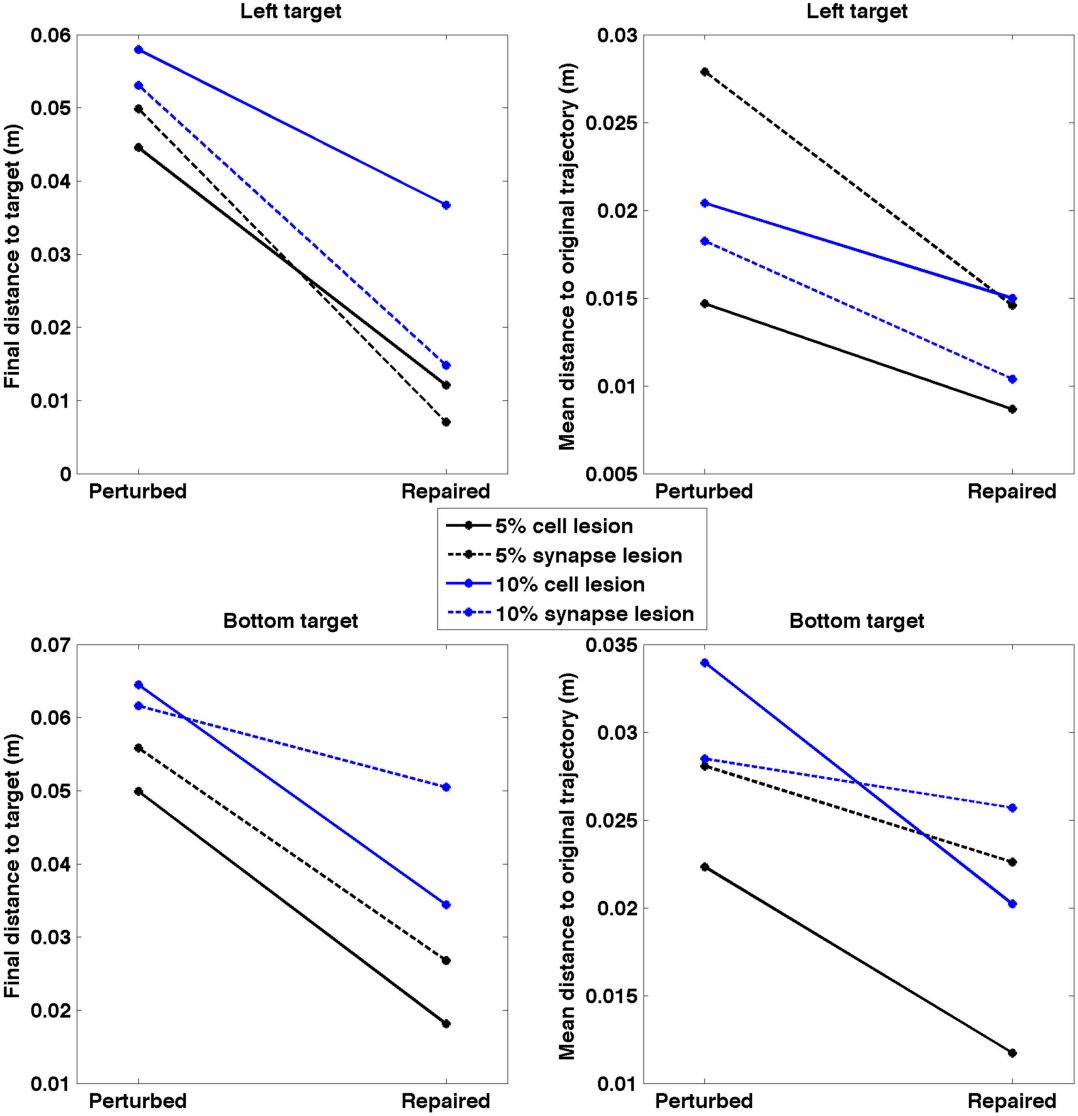
**Quantitative measures of repair neurostimulation**. Both metrics reflect a behavioral improvement after neurostimulation for all conditions. (Left) Final distance between hand and target. (Right) Mean point-wise distance to original arm trajectory.

After applying the repair neurostimulation to the lesioned network, the resulting motor activity yielded virtual arm reaching trajectories that more closely resembled the original trajectory (Figure 11). Both final distance to target at the end of the reaching trial, and the mean point-wise distance along the trajectory, were reduced after neurostimulation repair (Figure 12). The trajectories restored in the 5% lesion conditions were closer to the original than those restored in the 10% lesions conditions. Overall, mean distance to target was reduced from 5.47 to 2.51 cm after repair. Mean point-wise distance between trajectories was reduced from 2.43 to 1.61 cm. The same dataset was used to calculate the arm trajectories (Figure 11), behavioral metrics (Figure 12), and spike train metrics (Figure 9). This provides a direct link between neural activity and reaching behavior, thus facilitating the interpretation of the results.

## 4. Discussion

We implemented a neurocontroller using kernel adaptive filtering techniques on spike trains, which produced neurostimulation patterns that restored neural and functional responses in a lesioned biomimetic spiking network model. Neurostimulation partly restored the behavioral performance of a realistic virtual musculoskeletal arm which was driven by the network, allowing it to reach close to the original trained targets through trajectories similar to those produced before lesioning. The neurocontroller was able to compensate for 2 different types of lesions: a cell death and synaptic loss model. Neurodegenerative and ischemic (stroke) disease (Lytton et al., 1999) may be a cause of cell death. Certain neurodegenerative disease, such as Alzheimer's (Palop and Mucke, 2010; Rowan et al., 2014), as well as traumatic brain injury (Gupta and Przekwas, 2013), have been shown to cause both cell death and synaptic loss. This work also serves as a proof of concept to underline the potential benefits of neural simulations to evaluate neurocontrollers. These include the ability to reproducibly generate the required probing datasets, and detailed access to all effects of neurostimulation on the system, ranging from cells and synapses to virtual arm muscle electromyogram (EMG) or arm position. As discussed in the next subsection, further work is needed to increase the similarity between model and real brains and pave the way toward clinical applications.

One interesting commonality of the neurocontrol solutions for different directions of reach and types of damage was the synchrony with respect to the original spiking oscillations. Coordinated oscillations within and across brain areas are a dramatic feature of brain activity noted in electrocorticography (ECoG), which must be reconciled with the feedforward nature of many tasks. Coordination of firing may well play an important role in feedforward systems as coordinated activation across multiple convergent units would provide strong drive by spatial summation. This provides an important complement to the efficacy of temporal convergence from repetitive firing of presumed labeled-line rate-coding units. Spike timing plays an important role in motor control, as evidenced by studies demonstrating precise spike synchronization is involved in the preparation and execution of movement (Riehle et al., 1997; Grammont and Riehle, 1999; Rubino et al., 2006). Our bin time analysis indicated that, for some conditions, shorter time scales resulted in higher PETH correlation, supporting the importance of precise spike timing. However, for other conditions, longer time scales resulted in higher correlations, suggesting firing rate played a predominant role in neural coding. These longer time bins were also consistent with the sliding window duration (50 ms) used to compute the motor output commands from the EM population. Elucidating the exact role that the different time scales play will require further data and analysis.

Overall, we found the 10% lesions more difficult to fully restore than the 5% ones. Presumably, given the highly recurrent network connectivity, it was harder to find unaffected polysynaptic pathways to reproduce original activity in the more severe condition. This could be further explored from a directed graph-theoretic perspective by looking at numbers and types of remaining motifs in the context of removing nodes and edges. We also note that a physiological system would have continued synaptic plasticity so that the brain would be learning at the same time as the neurocontroller is learning, a phenomenon known as co-adaptation. This effect could be incorporated into our model by continuing the learning mechanisms in the network model during the period of neurostimulation (Song et al., 2013; Rowan et al., 2014), thereby providing some of the long-term neural plasticity induced by neurostimulation. Studies have shown motor cortex plasticity aids in motor function recovery after injury (Kleim et al., 2003; Jackson et al., 2006; Ramanathan et al., 2006), and the development of neurocontrollers will allow more precise deployment of plasticity-inducing stimulation therefore leveraging its rehabilitative effects.

Employing a biomimetic neuronal network to control a biomimetic virtual arm provides a matching of (relatively slow) dynamics that differs greatly from control of a simpler kinematic 2-link arm or a mechanical robotic arm (Dura-Bernal et al., 2015b). This matching of biological verisimilitude also offers the opportunity to understand control in terms of specific muscle contractions that can be compared to clinical cases, as the effectors in the model provide muscle activation rather than control of joint angle. Similarly, the sensory afferents measure muscle length and therefore correspond to the muscle spindle proprioceptors embedded in muscle.

As expected, there are many potential network solutions that can be drawn upon to produce a particular arm trajectory—this is an extremely high dimensional neural system being applied to a lower dimensional virtual arm. In the context of neural Darwinism (Edelman, 1987), our neurocontroller is able to choose, from among these multiple adapted (fit) neural subsystems, ones that are also able to solve the problem in the absence of the original full system. In neural Darwinism, this concept is referred to as neural degeneracy. The same concept also arises in consideration of echo state networks. From this perspective, pieces of these potential systems are selected during the initial probing phase of development of the inverse controller and a full dynamics is then drawn from the population of these dynamical fragments. Further study might enable us to map the fragments that were used in the solution in order to generate an explicit subspace of primitives both at neural firing and muscle synergy levels.

### 4.1. Limitations and challenged ahead

Our study provides groundwork for the novel application of kernel adaptive filtering methods to neural control, and evaluation of this approach via biomimetic brain models. Our model includes a number of biologically-realistic features, including intrinsic spiking properties for different cell types, cortex-based connectivity or neural oscillations. This level of detail is higher than that of many neural models, such as recurrent neural networks (RNNs), which have been shown to be useful to investigate neural circuit mechanisms underlying cognitive function (Mante et al., 2013; Sussillo et al., 2015). However, translation of this work to clinical applications will requires advancing many aspects. The realism and level of detail of the brain and neurostimulation models need to escalate drastically. The neurocontroller needs to be adapted to robustly exploit the dynamic, incomplete and complex data recorded from the brain. Progress in neural recording and stimulation technologies will also be critical to gradually move toward the clinical domains.

Our cortical model differs from the real brain in many ways, which should be considered when interpreting the results. Our model assumes a controlled scenario with full reproducibility of motor outputs and responses to neurostimulation. This strongly contrasts with the high variability and limited reproducibility in real brains. Our simulation captures several hundred neurons in a single cortical area. In reality, sensorimotor tasks likely involve millions of neurons from many regions (thalamus, basal ganglia, cerebellum, sensorimotor cortices, …) firing in coordinated patterns across and within areas (Douglas and Martin, 2012). The few cell types that we model as point neurons are only a minuscule fraction of the hundreds of cell categories that have been identified (Harris and Shepherd, 2015), each with distinct physiological properties and intricate dendritic and axonal morphologies. Our population-based connectivity matrices are far from capturing brain connectivity, which ranges from the subcellular patterns of synapses along dendrites, to laminar microcircuitry, to long range inter-areal connections. The recent full 3D reconstruction of a microscopic volume of cortical tissue evidenced its extraordinary complexity: 193 dendrites, 1407 axons and 1700 synapses were identified in a 40 × 40 × 50 micrometer volume (approximate size of a single cell body) (Kasthuri et al., 2015). These circuits provide the neural substrate for a myriad of neural coding and computation principles. Understanding and including them in our models is key to bridging the gap between neural activity and perception, cognition or behavior. Linking to behavior also requires more accurate models of the periphery systems, including the spinal cord (Alstermark and Isa, 2012) and motor plant (Loeb and Tsianos, 2015).

Large scale international efforts such as the Human Brain Project or the BRAIN initiative have fueled progress in computational neuroscience. As a result, brain models can incorporate and mimic anatomical and physiological data with an unprecedented level of detail. The model by Potjans and Diesmann (2014) with 80,000 point neurons and 0.3 billion synapses integrated a large body of cell type and connectivity data and reproduced many dynamical properties of cortical microcircuits. More recently, cellular and synaptic organization principles derived from experimental data were used to build what has been labeled as “the most complete simulation of a piece of excitable brain matter to date” (Koch and Buice, 2015; Markram et al., 2015). Cell models were classified into 207 types with distinct electro-physiological and full 3D morphological reconstructions, derived from recording and labeling over 14,000 neurons. The full simulation, which consisted of 31,120 neurons and 37 million synapses occupying approximately the size of a cortical column, enabled studying dynamic interactions across the molecular, cellular and circuit levels. These models, however, still lack direct links to behavior, as well as learning mechanisms, such as STDP or reinforcement learning.

We are similarly working on extending our cortical spiking model to include over 10,000 cells, 0.5 million synapses, 6 cortical layers, spinal cord circuits, and input from premotor cortex which mediates target selection (Chadderdon et al., 2014; Dura-Bernal et al., 2015a). In collaboration with experimentalists, we are also fully characterizing the 3D morphology and electrophysiology of the two main types of pyramidal cells in motor cortex (corticostriatal and corticospinal) (Suter et al., 2013; Neymotin et al., 2015). These will be embedded in the network simulations in order to study the multiscale dynamics linking molecular and cellular processes (McDougal et al., 2013) to the circuit and information processing level (Lytton et al., 2014; Marcus et al., 2014). Applying the neurocontrol approach developed in this paper to the increasingly detailed brain models would be an interesting step toward building practical clinical applications.

At the same time, we need to develop more realistic models of the effects of neurostimulation, which in our model is limited to increasing external inputs to the cells. This can be achieved for example by adding the optogenetic channelrhodopsin channel to the cell model (Ching and Ritt, 2013; Kerr et al., 2014), or by characterizing the recruitment of neurons during intracortical extracellular microstimulation (Overstreet et al., 2013; Hartmann et al., 2015). It is also important to comprehensively model the collateral effects of electric field stimulation, since studies have found that fibers of passage get preferentially excited (McIntyre et al., 2004), potentially leading to undesired effects.

Future improvements to the proposed neurocontroller will focus on its ability to generalize and employ different types of probing data. The inverse mapping assumes that the solution is spanned by the basis formed by the probing patterns. As we increase the complexity of the model and the severity of the lesions to repair, solutions interpolated from a limited number of probes may not effectively address the richness of the circuit dynamics. A possible extension would be to implement multiple iterations, such that the spiking network output after stimulation is fed back as input probing data to the neurocontroller. Another option that could improve performance is to include probing data with a larger spectrum of outcomes, as well as with different timescales. Local field potentials (LFPs) or ECoG signals are interesting candidates, and are closer to the type of probing data that could be obtained from real brains. Similarly, the mapping could be made directly between stimulation and motor responses, such as arm kinematics or muscle activations, leading to solutions with optimal behavior performance but potentially different neural patterns. Although this is an interesting option, an advantage of directly targeting brain signals is that it could potentially be applied in scenarios (e.g., sensory or cognitive dysfunctions) where the target behaviors cannot be clearly specified or are not available.

The neurocontroller described here requires applying very precise spatiotemporal stimulation, at the single-cell and millisecond resolution. Recent studies (Warden et al., 2014) demonstrate this is already possible with optogenetic stimulation, which was, for example, used to activate single place-cells in hippocampus (Rickgauer et al., 2014). High-resolution stimulation was also able to bring retinal prosthetic capabilities closer to normal vision, by optogenetically stimulating 9800 ganglion cells (Nirenberg and Pandarinath, 2012). Further advancements will enable more selective cell targeting and larger number of simultaneous stimulated cells (Suter et al., 2014).

The latest developments in brain-machine interfaces (Miranda et al., 2015) aim to build the next generation of implantable closed-loop neuroprosthetics, with applications including memory restoration (Hiscott, 2014) or treatment of neuropsychological disorders (Nelson and Tepe, 2014). Combining neuroprosthetics with biomimetic brain models has the potential to leverage the system's performance: the simulated circuits can interact directly with the biological brain circuits (Tessadori et al., 2012; Lee et al., 2014) and complement neurocontrol methods with biological mechanisms of co-adaptation and learning to achieve the target functional task (Sanchez et al., 2012; Kocaturk et al., 2015).

Spinal cord stimulation mediated by neurocontrol methods has successfully been employed for motor function restoration (Nishimura et al., 2013; Grahn et al., 2014). Intracortical stimulation to motor cortices has largely been limited to probing and understanding the elicited responses. However, an accumulating body of evidence (Jackson et al., 2006; Arle and Shils, 2008; Jefferson et al., 2015) suggests it could have far-reaching neurorestorative applications if coupled with appropriate neurocontrol methods. Biomimetic brain models may provide a useful tool to develop, evaluate and implement these neurocontrollers.

## Author contributions

All authors listed, have made substantial, direct and intellectual contribution to the work, and approved it for publication.

### Conflict of interest statement

The authors declare that the research was conducted in the absence of any commercial or financial relationships that could be construed as a potential conflict of interest.
